# Enriched environment causes epigenetic changes in hippocampus and improves long-term cognitive function in sepsis

**DOI:** 10.1038/s41598-022-14660-6

**Published:** 2022-07-07

**Authors:** Emily Córneo, Monique Michels, Mariane Abatti, Andriele Vieira, Renata Casagrande Gonçalves, Filipe Fernandes Gabriel, Heloisa Borges, Amanda Goulart, Natan da Silva Matos, Diogo Dominguini, Roger Varela, Samira S. Valvassori, Felipe Dal-Pizzol

**Affiliations:** 1 Laboratory of Experimental Pathophysiology, Graduate Program in Health Sciences, University of Southern Santa Catarina (UNESC), Av. Universitária, 1105, Criciúma, SC 88806000 Brazil; 2 Translational Psychiatry Laboratory, Graduate Program in Health Sciences, University of Southern Santa Catarina (UNESC), Criciúma, SC Brazil

**Keywords:** Biochemistry, Immunology

## Abstract

Sepsis is defined as a life-threatening organ dysfunction caused by an inappropriate host response to infection. The presence of oxidative stress and inflammatory mediators in sepsis leads to dysregulated gene expression, leading to a hyperinflammatory response. Environmental conditions play an important role in various pathologies depending on the stimulus it presents. A standard environment condition (SE) may offer reduced sensory and cognitive stimulation, but an enriched environment improves spatial learning, prevents cognitive deficits induced by disease stress, and is an important modulator of epigenetic enzymes. The study evaluated the epigenetic alterations and the effects of the environmental enrichment (EE) protocol in the brain of animals submitted to sepsis by cecal ligation and perforation (CLP). Male Wistar rats were divided into sham and CLP at 24 h, 72 h, 10 days and 30 days after sepsis. Other male Wistar rats were distributed in a SE or in EE for forty-five days. Behavioral tests, analysis of epigenetic enzymes:histone acetylase (HAT), histone deacetylase (HDAC) and DNA methyltransferase (DNMT), biochemical and synaptic plasticity analyzes were performed. An increase in HDAC and DNMT activities was observed at 72 h, 10 days and 30 days. There was a positive correlation between epigenetic enzymes DNMT and HDAC 24 h, 10 days and 30 days. After EE, HDAC and DNMT enzyme activity decreased, cognitive impairment was reversed, IL1-β levels decreased and there was an increase in PSD-95 levels in the hippocampus. Interventions in environmental conditions can modulate the outcomes of long-term cognitive consequences associated with sepsis, supporting the idea of the potential benefits of EE.

## Introduction

Sepsis and septic shock are potentially life-threatening conditions that are associated with high morbidity and mortality^1,2^. A relevant characteristic of sepsis is the presence of encephalopathy followed by microglial activation^3^ and the consequent expression of inflammatory mediators (cytokines and chemokines) and oxidative stress^4–8^. Recent studies show how oxidative stress insults and inflammatory events can lead to the vulnerability of the expression of some genes and epigenetic alterations^9–12^.

**Figure 1:**
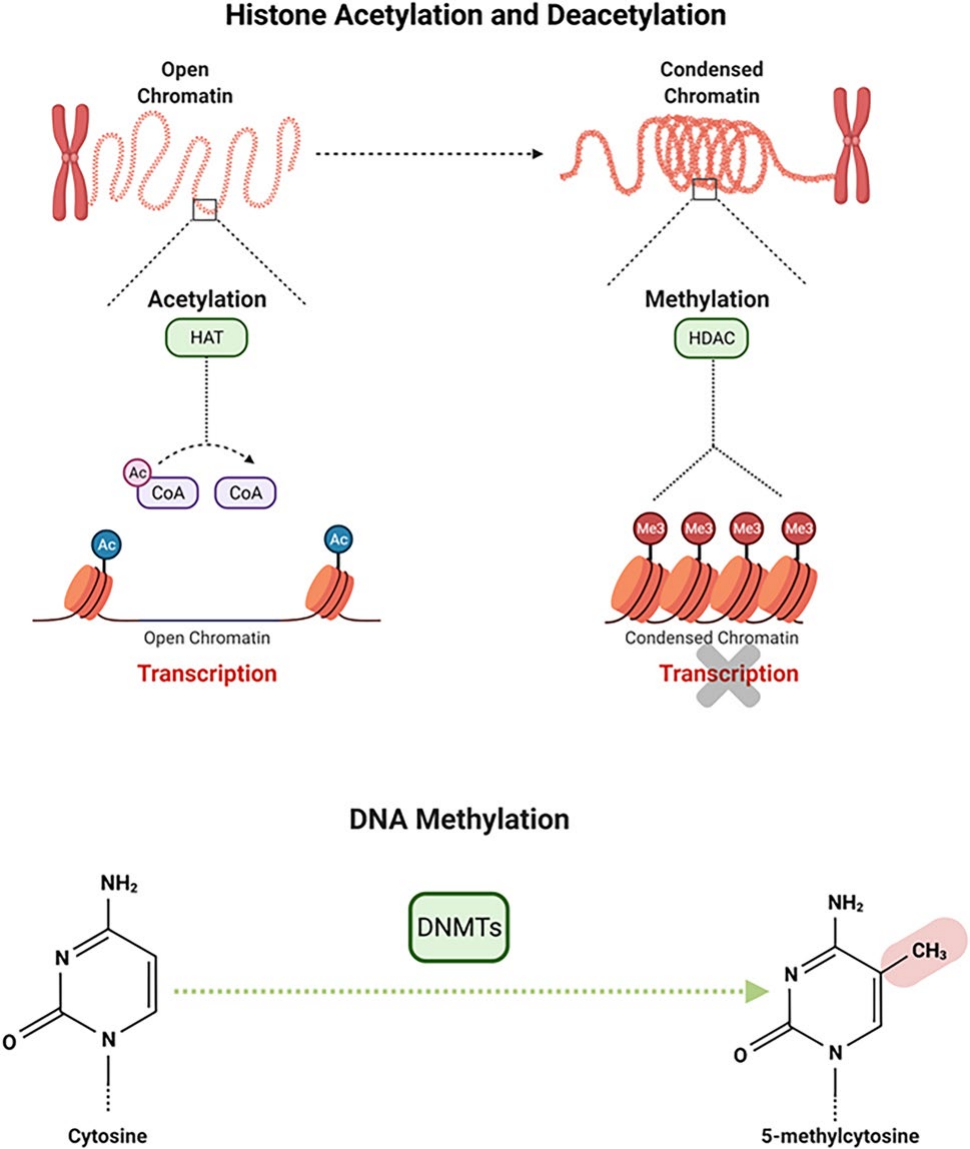
Histone acetylation and deacetylation through HAT and HDAC enzymes respectively.

Changes in gene expression during sepsis initiation and progression are related^13–15^. Epigenetic mechanisms are considered flexible parameters and can be altered by various stimuli, such as stress and changes in the environment. Failures in the establishment or maintenance of epigenetic marks can result in improper activation or inhibition of several genes and alter normal cell physiology leading to the appearance of pathologies^16,17^. The main epigenetic regulations are related to the dynamic states of chromatin, such as changes in histones and in the DNA methylation pattern. Histones are small proteins that, by complexing with DNA, form the nucleosome core. These proteins can be in one of two different forms, acetylated or deacetylated, which are regulated by the enzymes histone acetylases (HATs) and histone deacetylases (HDACs). Histone acetylation increases genic transcription while histone deacetylation represses it^18^. In addition, epigenetic modifications of DNA also regulate genic transcription, such as DNA methylation. Methylation of DNA involves covalent binding of a methyl group to cytosines by enzymes called DNA methyltransferases (DNMTs), which lead to promoter methylation and gene transcription is suppressed^19^ (Fig. 1).

Environmental conditions play a fundamental role in the cognitive performance of several neuropsychiatric diseases, affecting in a positive or negative way, depending on the stimulus that this environment offers and are considered factors that can modulate epigenetic mechanisms^20,21^. The environment considered standard (SE) is one that offers reduced sensory and cognitive stimulation, whereas the enriched environment (EE) is defined as an environment that presents physical and social stimuli^22,23^. The negatively altered behavioral and neuroendocrine response is seen in a standard setting^21,24^. Evidence shows that patients admitted to the ICU are subject to this environment, which may have negative effects on cognitive performance^25,26^. However, an EE can improve spatial learning, decrease mortality and prevent cognitive deficits induced by stress from a certain pathology^22,27^.

Current animal studies show that neurocognitive shifts were accompanied by significant changes in biomarkers of the immune response and hippocampal synaptic plasticity^28–30^. Thus, it is understood that the effects of environmental conditions on cognitive function are reversible, with an improvement in cognitive performance when animals are governed in an EE^28^.

Epigenetic changes can provide mechanistic information about the occurrence and treatment of diseases, but little is known about these mechanisms during the development of sepsis in the brain. Here, epigenetic changes in the hippocampus of animals submitted to the sepsis model have been reported and we suggest that interventions in environmental conditions may modulate the results of the long-term cognitive consequences associated with sepsis, supporting the idea of the potential benefits of an EE.

## Methods

### Ethics

The experimental procedures involving animals were carried out in accordance with the ARRIVE guidelines and the Brazilian legislation on animal welfare, with the approval of the Ethics Committee on the Use of Animals of the University of the Extreme South of Santa Catarina—Unesc (Protocol No. 008-2017/2).

### Animals

The study was divided into two experiments. Adult male Wistar rats, 60 days old, weighing between 200 and 300 g were used. The rats were kept in light–dark cycles of ± 12 h (7:00 am to 7:00 pm) at a temperature between 18 and 22 °C, relative humidity between 55 and 65%. The animals had free access to water and food. At the end of the experiments, the animals were euthanized by the decapitation method.

**Figure 2:**
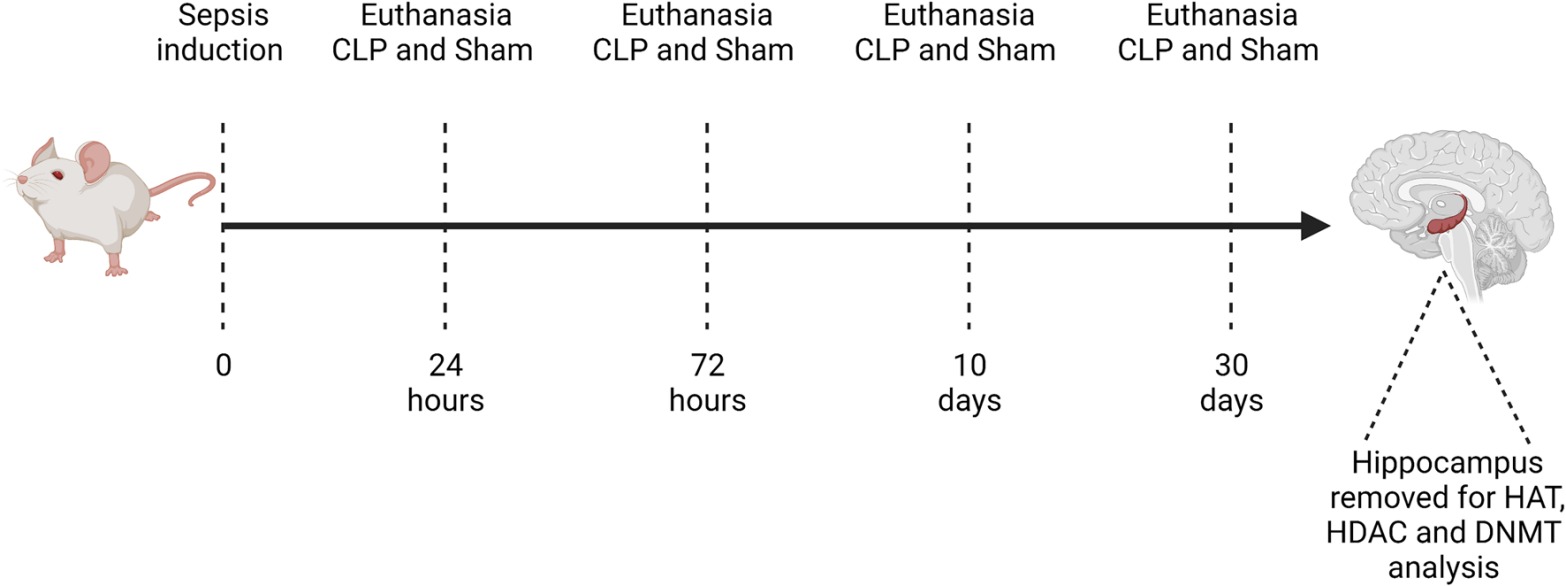
Timeline of the first experiment, dates of sepsis induction, animal euthanasia and structure removed for analysis.

The first part of the experiment was performed with 80 Wistar rats, which were divided into eight groups, and in each group the animals were euthanized at a different time to analyze the epigenetic changes. 1) Sham 24 h (nº = 7); 2) 24 h CLP (number = 13); 3) Sham 72 h (nº = 7); 4) CLP 72 h (nº = 13); 5) Sham 10 days (nº = 7); 6) CLP 10 days (nº = 13); 7) Sham 30 days (nº = 7); 8) CLP 30 days (nº = 13). According to the time delimited in each group, the animals were euthanized by beheading and the hippocampus was removed to analyze the activity of the enzymes HAT, HDAC and DNMT (Fig. 2).

**Figure 3:**
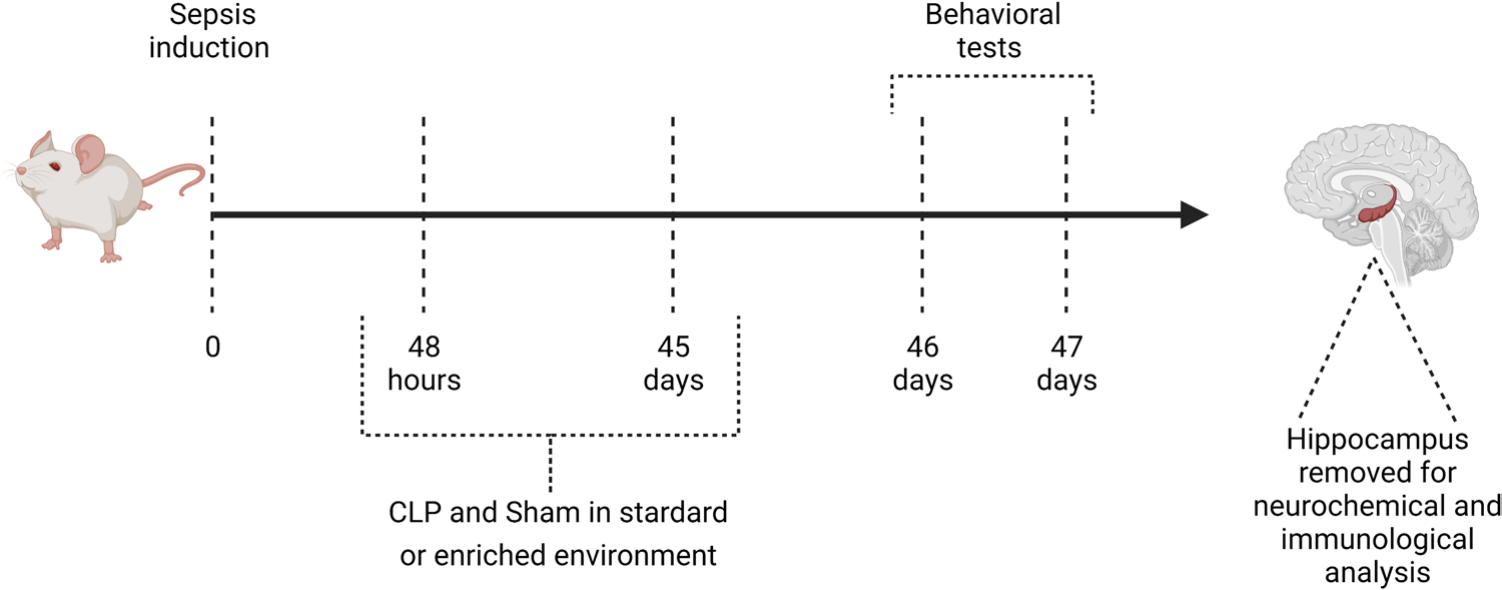
Timeline of the second experiment, date of sepsis induction, behavioral tests and structure removed for analysis. The animals were placed in cages at the university vivarium for behavioral tests.

The second experiment 46 Wistar rats were used, divided into four groups: (1) Sham + EE (nº = 7); (2) Sham + SE (nº = 7); (3) CLP + EE (nº = 16); 4) CLP + SE (nº = 16). After 48 h, the animals were distributed in a SE or EE and kept in these housing conditions for 45 days. After this period, on days 46 and 47, the animals were placed in white plastic cages (length 30 cm x width 20 cm × height 13 cm), consisting of a metal grid with an opening for offering a standard diet for rodents (composite of ground whole corn, wheat bran, soy bran, calcium carbonate, dicalcium phosphate, sodium chloride, amino acid, vitamin mineral) and water with free access, in the university vivarium. Subsequently, behavioral tests were performed and, finally, euthanasia by the decapitation method, with the removal of the hippocampus for subsequent neurochemical and immunological analysis (Fig. 3).

### Sepsis induction—cecal ligation and perforation (CLP) model

Male *Wistar* rats were subjected to CLP as previously described^31^. Briefly, animals were anesthetized using a mixture of ketamine (80 mg/kg) and xylazine (10 mg/kg), given intraperitoneally. Under aseptic conditions, a 3 cm midline laparotomy was performed to expose the cecum and adjoining intestine. The cecum was ligated with a 3.0 silk suture at its base, below the ileocecal valve, and was perforated once with a 14-gauge needle. The cecum was then squeezed gently to extrude a small amount of feces through the perforation site. The cecum was then returned to the peritoneal cavity, and the laparotomy was closed with 4.0 silk sutures. Animals were resuscitated with saline (NaCl 0.09%, 50 mL/kg) subcutaneously (s.c.) immediately after and 12 h after CLP. The same investigator always performed the CLP procedure to minimize variability between different experiments. The mortality of this model is around 40% that is consistent with severe sepsis. As control of the experimental design, the animals will be submitted to laparotomy without binding or perforation of the cecum (sham). All animals received antibiotics (ceftriaxone at 30 mg/kg) and dipyrone (80 mg/kg) every 6 h s.c. for 3 days, to prevent animal mortality^68^.

### Environmental conditions

After recovery from sepsis-inducing surgery (48 h), the animals were randomly assigned to two different environmental conditions: SE or EE and kept in these housing conditions for forty-five days. The SE consisted of common cage housing (41 cm × 34 cm × 18 cm), while the EE consisted of larger cages (80 cm × 45 cm × 22 cm) containing a small house and a racing wheel for voluntary exercise and toys that were changed three times a week with new toys of different shapes and colors. The SE animals were housed by group of five and the EE animals were housed by group of ten^28^.

#### HAT, HDAC, DNMT activity

At determined times (24 h, 72 h, 10 and 30 days), the animals were killed by decapitation, and the hippocampus was dissected (n = 5). The obtained samples were flash-frozen and stored at − 80 °C until nuclear proteins could be extracted. The hippocampus was subjected to a nuclear extraction protocol using a commercial Nuclear Extraction kit (Chemicon, USA). Nuclear extraction was used to evaluate the levels of HAT, HDAC, and DNMT activity.

In order to evaluate the activity of HAT, HDAC and DNMT, the nuclear extracts were submitted to an assay for the evaluation of HAT, HDAC, and DNMT activity using assay kit (colorimetric detection) according to the manufacturer's instructions (Epigentek cod. HAT P-4003-96; HDAC P-4002-96; DNMT P-3001-2). A standard curve was performed with serial dilutions of subtracts from each kit and positive and negative controls were added to the plate. Colorimetric reading was performed on an ELISA plate reader. The calculation of enzymes activity was performed based on the standard curve, and the values were presented in nM/μg of protein. Protein dosages were determined by the method of Lowry^31^ and bovine serum albumin was used as a standard.

### Behavioral tasks

After enriched environment protocol, the animals were subjected to habituation to the open-field task and the object recognition test. After the behavioral tests, the animals were sacrificed by decapitation, the hippocampus was immediately isolated on dry ice and stored at −80 °C for other analysis.

### Open-field test

Behavior was assessed in an open-field apparatus to evaluate both locomotor and exploratory activity. The apparatus was a 40 × 60 cm open field surrounded by 50-cm-high dark grey walls and a glass front wall. Black lines divided the floor of the open field into nine rectangles. Each animal was gently placed in the center of the open field and was left to explore the arena for 5 min (training session). The number of crossings (i.e., the number of times that each animal crossed the black lines, an assessment of locomotor activity) and rearing movements (i.e., the exploratory behavior observed in rats subjected to a new environment) were measured. Immediately after this procedure, the animals were taken back to their home cage. Twenty-four hours later, they were subjected to a second open-field session (test session). In both sessions, the number of crossings and rearings was counted during a 5-min period. A reduction in the number of crossings and rearings between the two sessions was considered as a measure of the retention of memory. The same experimenter, who was blind to group allocation, performed all the behavioral testing and manual scoring^33^. The experimental box was thoroughly cleaned with 70% ethanol between testing sessions.

### Object recognition

For object recognition animals were allowed to explore an open field. Training was conducted by placing rats in the field in which two identical cubes (objects A1 and A2) were positioned. Twenty-four hours post-training animals were allowed to explore the field in the presence of the familiar object A but a novel object C (a sphere with a square-shaped base). A recognition index was calculated and reported as the ratio TB/(TA + TB) (TA = time spent exploring the familiar object A; TB = time spent exploring the novel object B)^34^.

### Assessment of the concentration of IL-1β

Briefly, the hippocampus was homogenized in PBS buffer (100 mg of tissue per 1 mL). The levels of IL-1β were determined using commercially available enzyme-linked immunosorbent assays (ELISA), following the instructions supplied by the manufacturer (DuoSet kits, R&D Systems, Minneapolis, MN, USA). The results are expressed as pg/mL.

### Synaptic protein levels

Levels of synaptophysin (a specific synaptic vesicle marker that plays an important role in the release of neurotransmitters) were determined in the hippocampus using the commercial enzyme-linked immunosorbent assay kit (ELISA)^35^. To perform immunoblotting of PSD-95 *(postsynaptic density protein 95)*, tissue samples of hippocampus was homogenized in Laemmli buffer using beta-mercaptoethanol as a reductant (62.5 mM TrisHCl, pH 6.8, 1% (w/v) sodium dodecyl sulfate (SDS), 10% (v/v) glycerol) and equal amounts of protein (30 µg/well) were fractionated by polyacrylamide gel electrophoresis-sodium dodecyl sulfate (SDS-PAGE) and electro transferred to nitrocellulose membranes. The efficiency of the electro transfer was verified by Ponceau staining, and the membrane was then blocked in Tris-Tween buffer saline (TTBS: 100 mM Tris HCl, pH 7.5, containing 0.9% NaCl and 0.1% Tween 20) with 5% albumin. The membranes were incubated overnight at 4 °C with rabbit monoclonal anti-PSD-95 (1:1000). Secondary anti-rabbit IgG was incubated with the membrane for 2 h (1:10,000). The immunoreactivity was detected by chemiluminescence using ECL. Densitometry analysis of the films was performed using the ImageJ v.1.34 software. All results were expressed as a relative ratio between anti-PSD-95 and GAPDH.

### Statistics

The data from the behavioral test of habituation in the open field were evaluated through the mean and the standard error of the mean (EPM), being EPM the statistical difference calculated through the Student T test for independent samples. For the object recognition test, the results were expressed as median and interquartile range and the differences between training and test were evaluated by the Mann–Whitney U test followed by the Wilcoxon test.

For biochemical and immunological analyzes, data were expressed as mean ± standard deviation and compared with one-way analysis of variance (ANOVA) followed by the Tukey test. For the correlation of epigenetic enzymes, data were expressed using Pearson's correlation coefficient, considering the “r” value. Western blotting was quantified by the ImageJ ® program and presented as a ratio between protein and GAPDH. All tests were analyzed using SPSS version 20 and/or GraphPad Prism 5.0. In all analyzes, a level of *p* < 0.05 was adopted for statistical significance.

### Ethics approval and consent to participate

Animal studies were approved by the ethics commitment of our institutional ethics committee (Protocol No. 008-2017/2).

## Results

### Time-dependent alterations on HAT, HDAC and DNMT activities in hippocampus of animals submitted to sepsis.

**Figure 4:**
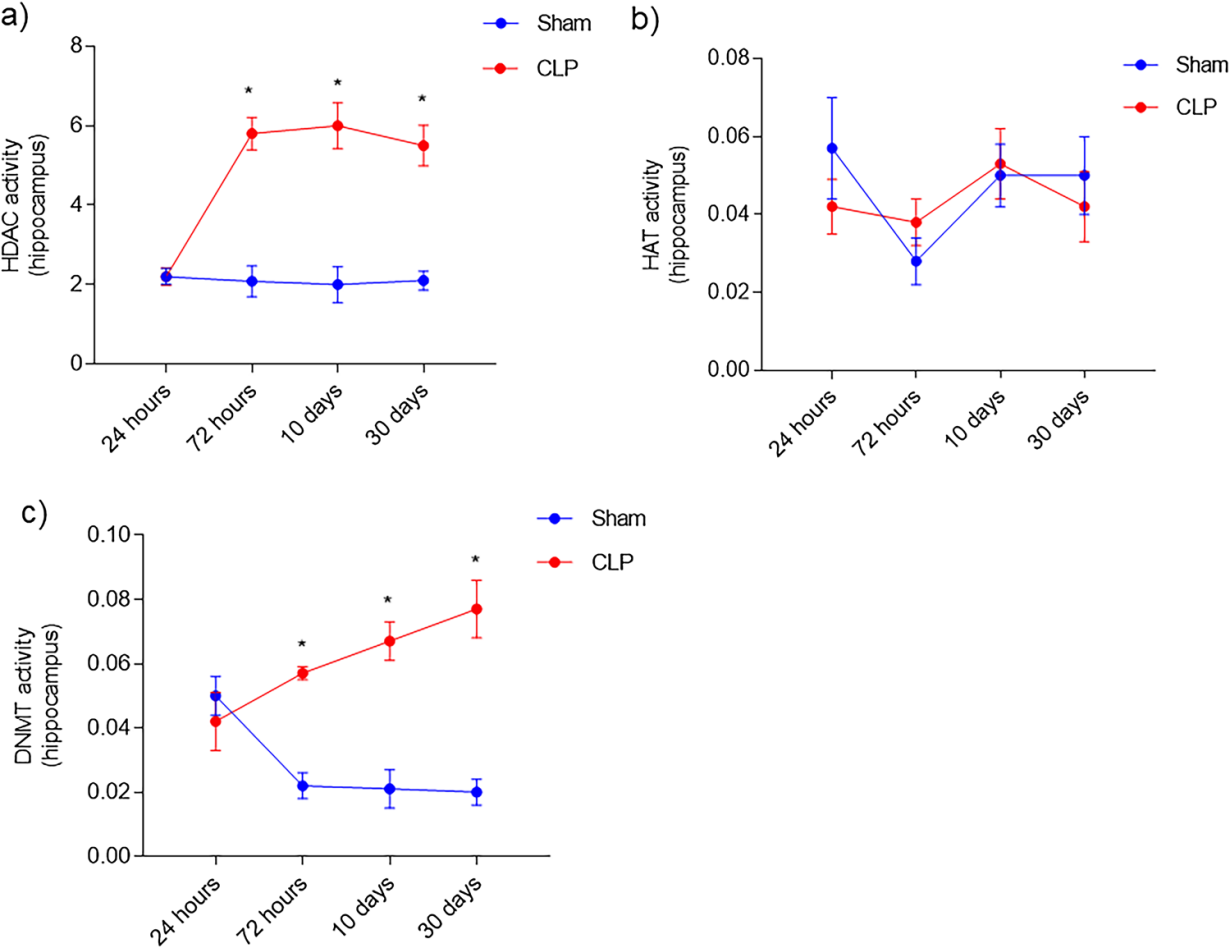
Activity of epigenetic enzymes HAT, HDAC and DNMT. HDAC, HAT and DNMT activities were measured in the hippocampus of animals submitted to sepsis at different times. HDAC (**a**); HAT (**b**) and DNMT (**c**). Data are expressed as mean ± SD and compared with one-way analysis of variance (ANOVA) followed by the Tukey test. nº = 6 each group. *p < 0.05 different from the CLP at the same time.

HDAC and HAT are enzymes associated with histone acetylation and deacetylation respectively. In HDAC activity, there was no difference at 24 h after sepsis (sham 24 h = 2.2 ± 0.2; CLP 24 h = 2.2 ± 0.22). However, from 72 h to 30 days (sham 72 h = 2.08 ± 0.39; CLP 72 h = 5.8 ± 0.41; sham 10 days = 2.0 ± 0.45; CLP 10 days = 6.0 ± 0.58; sham 30 days = 2.1 ± 0.24; CLP 30 days = 5.5 ± 0.51) after sepsis an increase in the activity of this enzyme was noted when compared septic to sham group (Fig. 4a). It was not observed changes in the activity of HAT enzyme in any time-point studied (sham 24 h = 0.057 ± 0.013; CLP 24 h = 0.042 ± 0.007; sham 72 h = 0.028 ± 0.006; CLP 72 h = 0.038 ± 0.006; sham 10 days = 0.05 ± 0.008; CLP 10 days = 0.053 ± 0.009; sham 30 days = 0.05 ± 0.01; CLP 30 days = 0.042 ± 0.009) (Fig. 4b). DNMT is associated with DNA methylation. There was no difference in DNMT activity 24 h after sepsis (sham 24 h = 0.05 ± 0.006; CLP 24 h = 0.042 ± 0.009). After 72 h, 10 and 30 days of sepsis induction there was an increase in the activity of DNMT (sham 72 h = 0.022 ± 0.004; CLP 72 h = 0.057 ± 0.022; sham 10 days = 0.21 ± 0.006; CLP 10 days = 0.067 ± 0.006; sham 30 days = 0.02 ± 0.004; CLP 30 days = 0.077 ± 0.009) (Fig. 4c).

### Correlation of DNMT and HDAC Epigenetic Enzymes

**Figure 5:**
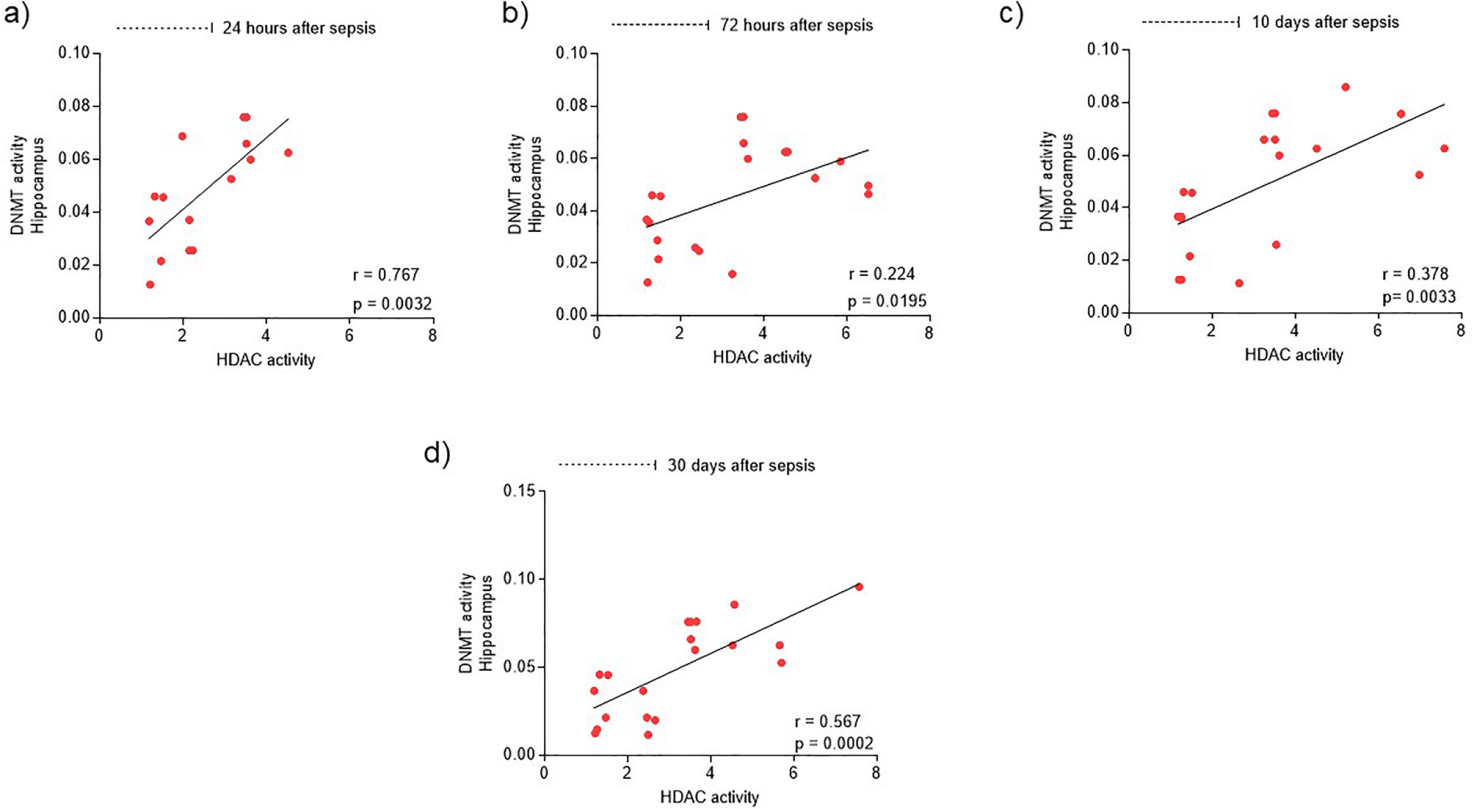
Correlation of the activity of epigenetic enzymes DNMT and HDAC in the hippocampus of animals submitted to sepsis. DNMT and HDAC activities were correlated in the hippocampus tissue of animals submitted to sepsis at different times. 24 h (**a**); 72 h (**b**) 10 days (**c**); 30 days (**d**). Data are expressed using Pearson's correlation coefficient, considering the value of “r”.

The correlation of the activity of epigenetic enzymes DNMT and HDAC was evaluated in the hippocampus of animals submitted to sepsis. It is understood that the DNMT enzyme is related to DNA methylation and the HDAC enzyme with changes in chromatin, and these factors interact with each other. The correlation is considered positive and significant when the points are not dispersed and consequently show a growing line. It was noticed that in the times of 24 h (r = 0.767; *p* = 0.0032) (Fig. 5a), 10 days (r = 0.378; *p* = 0.0033) (Fig. 5c), and 30 days (r = 0.567; *p* = 0.0002) (Fig. 5d), the activity of the two enzymes showed a significantly positive correlation. However, the 72-h time (r = 0.224; 0.0195) (Fig. 5b) showed a correlation, however, not significantly.

### Enriched environment effects on HAT, HDAC and DNMT activities in hippocampus of animals submitted to sepsis.

**Figure 6:**
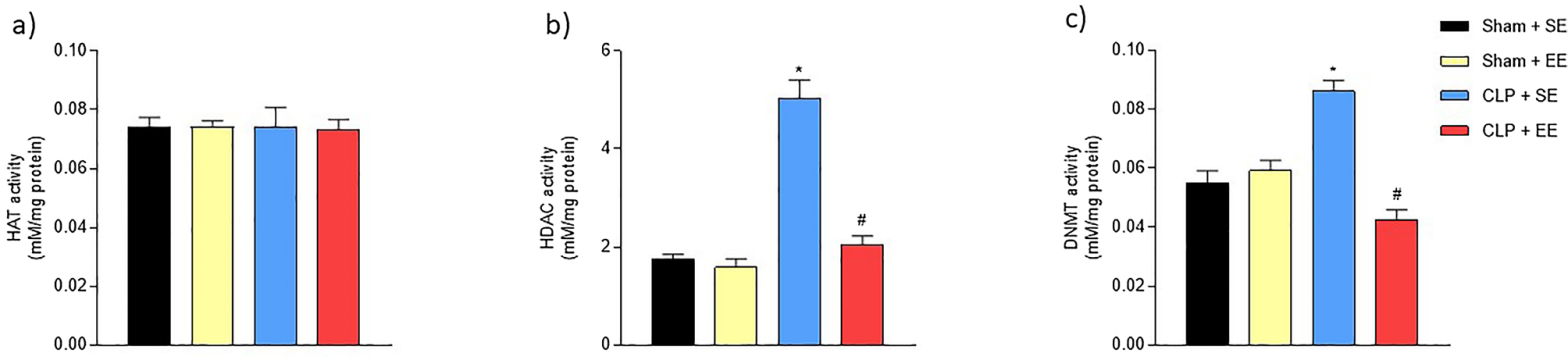
Effects of environmental enrichment on the activity of epigenetic enzymes in the hippocampus of animals subjected to sepsis. HDAC, HAT and DNMT activities were measured in the hippocampus of animals submitted to sepsis after the EE protocol (40-day period). HAT (**a**); HDAC (**b**) and DNMT (**c**). The data were expressed as mean ± SD and compared with one-way analysis of variance (ANOVA) followed by the Tukey test. nº = 6 each group. *p < 0.05 versus Sham + SE; #*p* < 0.05 versus CLP + SE. EE: environmental enrichment; SE: SE.

It was not observed changes in the activity of HAT enzyme after EE protocol (sham + SE = 0.074,125 ± 0.00,317; sham + EE = 0.074,100 ± 0.002,130; CLP + SE = 0.074,000 ± 0.006,825; CLP + EE = 0.73,100 ± 0.003,512) (Fig. 6a). In HDAC (sham + SE = 1.750,000 ± 0.099,247; sham + EE = 1.586,000 ± 0.173,136; CLP + SE = 5.032,000 ± 0.373,148; CLP + EE = 2.044,420 ± 0.178,453) and DNMT (sham + SE = 0.054,830 ± 0.004,216; sham + EE = 0.059,132 ± 0.003,548; CLP + SE = 0.086,234 ± 0.003,587; CLP + EE = 0.042,636 ± 0.003,278) enzymes activities there were an increase in CLP + SE group and in both enzyme activities, EE protocol reverse these alterations (Fig. 6b,c).

### Open-field test

**Figure 7:**
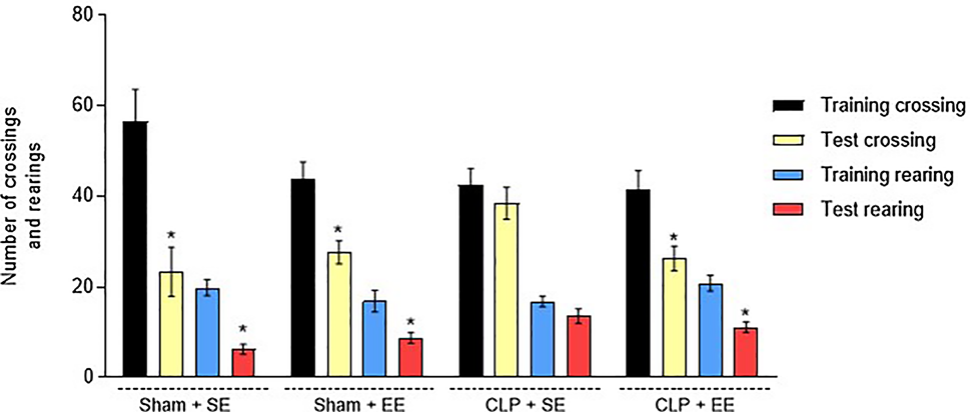
Behavioral test of exploratory and locomotor activity after the environmental enrichment protocol in animals submitted to sepsis. The bars represent mean ± SEM, the statistical difference being calculated using the Student T test for independent samples. nº = 10 each group. *p < 0.05 when comparing the test versus training of your own group. EE: environmental enrichment; SE: standard environment.

In order to assess habituation memory and impairment of animals, the habituation test in the open field was performed. The CLP + SE group showed cognitive impairment, as there was no significant difference between training and testing in exploratory (rearing) and locomotor (crossing) activity (CLP + SE: rearing training = 16.88 ± 1.172; rearing test = 13.63 ± 1.603; crossing training = 42.50 ± 3.655; crossing test = 38.50 ± 3.495). However, the CLP + EE group showed a significant difference between training and testing, (CLP + EE: rearing training = 20.91 ± 1.771; rearing test = 11.18 ± 1.174; crossing training = 41.45 ± 4.196; crossing test = 26.36 ± 2.684). demonstrating an improvement in habituation memory, confirming its ability to reverse the changes generated by sepsis (Fig. 7).

### Object recognition

**Figure 8:**
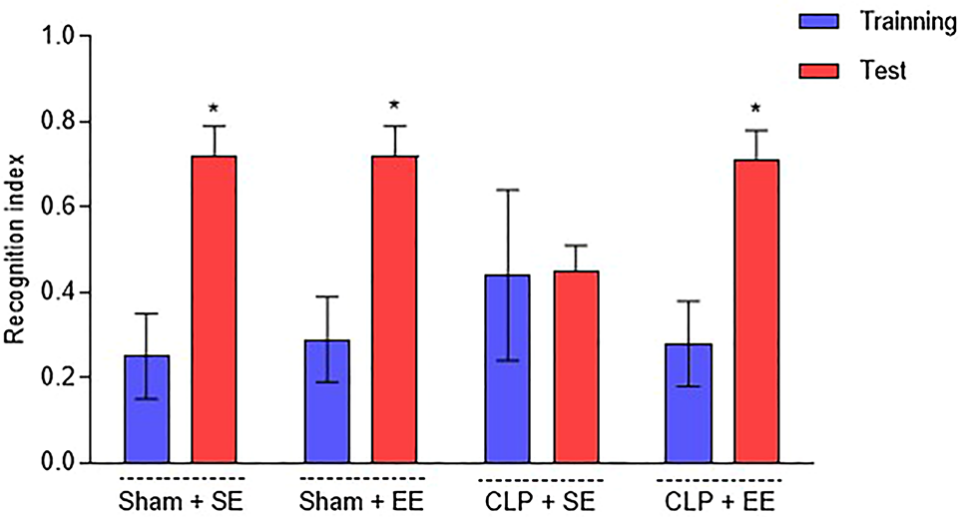
Object recognition task after the environmental enrichment protocol in animals submitted to sepsis. The recognition index is presented as median and interquartile ranges. Differences between training and testing were assessed using the Mann–Whitney U test followed by the Wilcoxon test. nº = 10 each group. *p < 0.05 when comparing the test versus training of your own group. EE: environmental enrichment; SE: standard environment.

To further explore the potential protective effect of the environmental enrichment protocol, the object recognition task was used to assess long-term memory. The CLP + SE group showed a memory deficit, as they did not present greater latency and remained exploring the new object (CLP + SE: training = 0.440 ± 0.200; test = 0.450 ± 0.060). However, the CLP + EE group showed a significant difference in the object recognition index between training and testing (CLP + EE: training = 0.280 ± 0.100; test = 0.710 ± 0.070), manifesting a long-term memory and confirming its ability to reverse the changes generated by sepsis (Fig. 8).

### IL-1β levels in hippocampus after enriched environment protocol in animals submitted to sepsis

**Figure 9:**
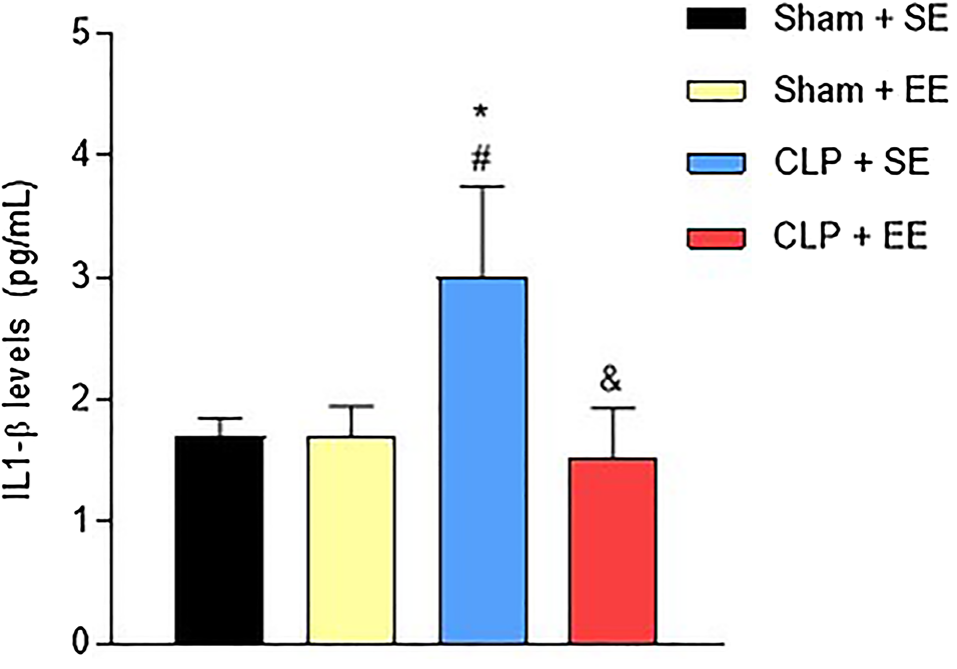
IL-1β levels in the hippocampus after the environmental enrichment protocol in animals submitted to sepsis. IL-1β levels were measured at the end of the environmental enrichment protocol in the hippocampus of animals subjected to sepsis. The data were expressed as mean ± SD and compared with one-way analysis of variance (ANOVA) followed by the Tukey test. nº = 6 each group. *p < 0.05 different from sham + SE; #*p* < 0.05 other than sham + EE; & *p* < 0.05 other than CLP + SE. EE: environmental enrichment; SE: standard environment.

IL-1β levels were analyzed in the hippocampus after the environmental enrichment protocol in animals submitted to sepsis. The CLP + SE group (3.00 ± 0.75) demonstrated high levels of IL-1β compared to the Sham + SE group (1.70 ± 0.15) and the Sham + EE group (1.70 ± 0.25). However, septic animals that were submitted to the environmental enrichment protocol (1.53 ± 0.40) had a significant decrease in IL-1β levels (Fig. 9).

### Synaptic protein levels

**Figure 10:**
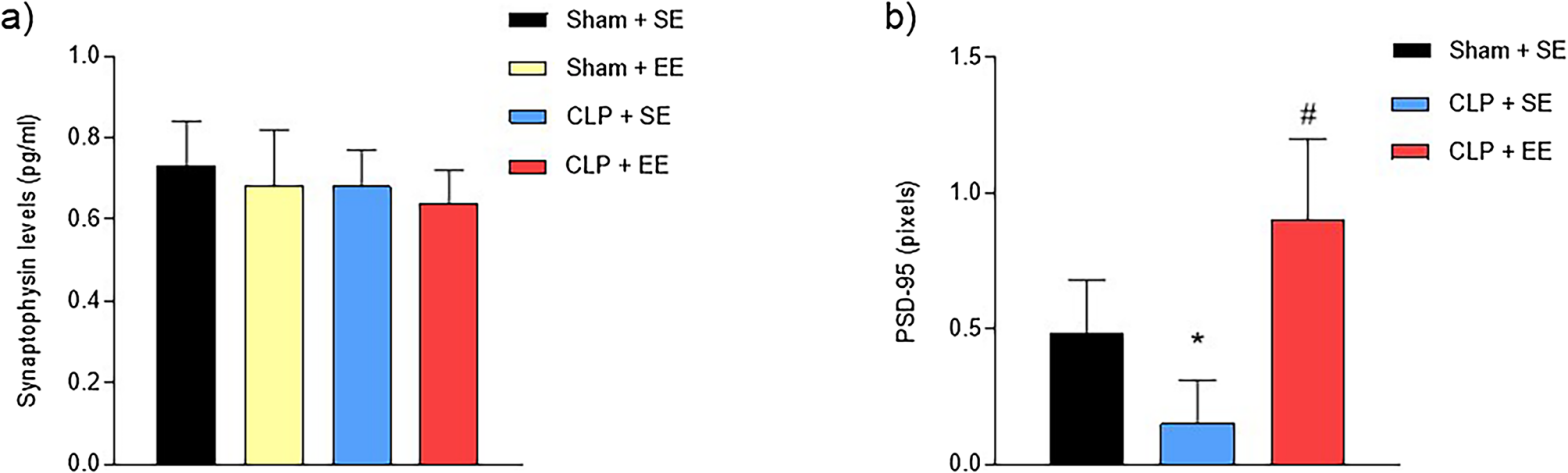
(**a**) Synaptophysin levels in the hippocampus after the environmental enrichment protocol in animals subjected to sepsis. Synaptophysin levels were measured at the end of the environmental enrichment protocol in the hippocampus of animals subjected to sepsis. The data were expressed as mean ± SD and compared with one-way analysis of variance (ANOVA) followed by the Tukey test. nº = 6 each group. (**b**) PSD-95 levels in the hippocampus after the environmental enrichment protocol in animals submitted to sepsis. PSD-95 levels were measured at the end of the environmental enrichment protocol in the hippocampus of animals subjected to sepsis. The data were expressed as mean ± SD and compared with one-way analysis of variance (ANOVA) followed by the Tukey test. nº = 6 each group. *p < 0.05 different from sham + SE; # different from CLP + SE. EE: environmental enrichment; SE: standard environment.

Synaptophysin is considered a specific marker of synaptic vesicles, which plays an important role in the release of neurotransmitters. There were no significant changes in synaptophysin levels in any of the groups analyzed after the EE protocol in animals submitted to sepsis (sham + SE = 0.73 ± 0.11; sham + EE = 0.68 ± 0.14; CLP + SE = 0.68 ± 0.09; CLP + EE = 0.64 ± 0.08) (Fig. 10a).

To determine the effect of the EE protocol on synaptic plasticity, a highly stable synapse structural protein, also known as PSD-95, was evaluated. The CLP + SE group (0.15 ± 0.16) showed a decrease in PSD-95 levels compared to the Sham + SE group (0.48 ± 0.20). However, the EE protocol (0.90 ± 0.30) was able to increase PSD-95 levels in the hippocampus of animals subjected to sepsis (Fig. 10b).

## Discussion

This study reported the presence of epigenetic changes in an animal model of sepsis, first at different times and later showed that exposure to an EE protocol can reduce these changes and decrease the deficits generated by the pathophysiology of sepsis.

HAT and HDAC are enzymes related to histone modification, more specifically acetylation and deacetylation of these proteins. Numerous enzymes act together to add or remove covalent modifications in histones, interacting with each other and with other mechanisms to maintain chromatin conformation and transcript control^36,37^. DNMTs are enzymes responsible for DNA methylation, they replace the cytosine H5 of DNA by a methyl group. Methylation of the gene leads to inhibition of transcription and it is related to gene silencing, in addition to leading to chromosomal instability^38^. When hypomethylation or hypermethylation occurs then an imbalance in gene expression occurs, leading to important consequences such apoptosis and exacerbated inflammation. In this study, there was no difference between groups in HAT activity. However, changes in HDAC and DNMT activities were notable. A significant increase in HDAC and DNMT activities was reported up to 30 days. These results suggest alterations in the genetic transcription in an animal model of sepsis, mainly related to silencing of gene expression up to 30 days, and this is related to cognitive impairment that we demonstrated previously^6,7^.

In vitro research suggests that epigenetic changes are essential for establishing the tolerance generated to the pathogen. In autoimmune diseases, cancer and neurological disorders, several epigenetic changes have been reported, which have been associated with the onset or worsening of diseases^39^. In addition, recent studies have demonstrated the involvement of epigenetic changes in targeting the macrophage phenotype, as well as in the function of dendritic cells during sepsis^40–42^.

Epigenetic regulation occurs through several mechanisms, but in general it is characterized as a regulated organization of gene loci in transcriptionally active or silent states^43^. Recent studies have shown that in postmortem analyzes of the brains of septic patients, the expression of the HDAC enzyme was high^44^. It is known that under normal conditions, the activity of the enzymes HAT and HDAC remains in balance. However, this pattern is modified at the beginning of the pathophysiology of sepsis, as there is an intense expression of pro-inflammatory mediators, making it necessary for the HAT enzyme to open the chromatin structure to transcript inflammatory genes, such as IL-1β, which is involved in the central nervous effects of sepsis^45,46^, as we showed here. This process ends up by HDAC enzymes, which can be activated or induced by bacterial compounds^47^, leading to the reconstitution of chromatin closely linked to the silencing of the gene^48^, which can be associated with immunosuppression^49,50^.

However, it is possible to correlate the activity of the enzyme HDAC and DNMT during the pathophysiology of sepsis, as these two epigenetic enzymes showed a positive correlation at the times of 24 h, 10 days and 30 days after sepsis. The interactions between DNA methylation and chromatin modification have been known and described for many years^9^. There are studies that already prove that methylated DNA can recruit HDAC enzymes via MeCP2 (methyl-CpG-binding protein 2), leading to histone deacetylation and chromatin condensation, consequently disfavoring transcriptional initiation^44^. Another study showed that the use of tricostatin A, an HDAC inhibitor, attenuated the DNA repression induced by its own methylation, pointing to a cross effect between these two epigenetic changes^38^.

Here we confirm that exposing Wistar rats to an EE was able regulated enzymes activities HDAC and DNMT in hippocampus. According to Lambert et al.^51^ EE can produce changes in the hippocampus related to learning and memory. EE is associated with neurochemical and physiological changes in the brain. The increase in dendridic ramifications, including in the pyramidal cortical II/III and V, synapses, neuronal size, neurogenesis, neurotropin expression and neurotransmitter release have been related to EE^52,53^.

Current studies demonstrate that HDACs are elevated in the brain of septic animals, showing their relationship in neurodegeneration and in the cognitive functions generated by sepsis^17^. Because of this, it is understood that epigenetic modulation is involved in the septic brain and the inhibition of HDACs can serve as a potential therapeutic approach for the treatment of sepsis-associated encephalopathy^50^.

The induction of sepsis in animals generates deficits in memory, learning and often exhibits symptoms of a depressive type^3,54^. It is known that when there is exposure to an environment characterized as a standard, these factors do not improve. The data from the present study show that septic animals submitted to a SE showed cognitive impairment and exhibited a long-term memory deficit when compared between their training and the test. It is known that the neurocognitive consequences generated by the pathophysiology of sepsis can perpetuate in a SE, but when there is exposure to an enriched environment, studies show that these consequences generated by the disease show improvements^28,55^. These findings are in agreement with the results evidenced in this study, since the septic animals submitted to the EE protocol obtained the ability to reverse the changes generated by sepsis, as they had a long-term memory. Notably, the role of environmental conditions on cognitive function is reversible, showing an improvement in cognitive performance when subjected to an EE protocol, which is characterized by leading to an increase in spatial learning and preventing cognitive deficits generated by sepsis^25,26,30^.

Evidence shows that DNA methylation is dynamically modulated by events dependent on neuronal activity^56,57^. DNMTs enzymes not only play a role in gene printing and transcription regulation in the early stages of CNS development, but are also indispensable in learning, memory and cognition, and there are recent reports that show the participation of DNMTs in consolidating the memory of long-term^58–60^.

It is known that IL-1β is considered an initial marker of sepsis, as it expresses pro-inflammatory effects that contribute to an intense cellular response to the pathogen, in addition to having the ability to release secondary mediators^61,62^. In the present study, IL-1β levels remained elevated in the hippocampus, forty-five days after sepsis. However, animals submitted to the EE protocol showed significantly decreased levels in brain structure. It is then understood that brain inflammation persists late in septic animals, but that EE has the ability to reverse these damages generated by the pathophysiology of sepsis.

Synapses are vulnerable in neurodegenerative conditions, so sepsis can destroy synaptic proteins and contribute to cognitive deficit^63^. PSD-95 is a highly stable synapse structural protein that binds to NMDA receptors and stabilizes them in postsynaptic neurons, participates in the maturation of synaptic buttons, and favors excitatory synapse^64,65^. Another signaling-dependent protein mentioned above is synaptophysin, a membrane protein present in synaptic vesicles, which can be used as a specific marker of synaptic vesicles present in the synaptic terminals of presynaptic neuron that plays an important role for neurotransmitter release^35,66^. The present study showed that sepsis decreases PSD-95 levels in the brain, with the hippocampus being the most affected. However, these levels increase in septic animals submitted to the EE protocol. Above all, the synaptophysin presynaptic protein was not affected in septic animals. A study by Moraes et al.^67^ exposed that synaptophysin did not show changes in its levels, however the PSD-95 protein demonstrated significantly reduced levels in the hippocampus of mice 24 h and 3 days after sepsis induction, however 30 days after sepsis the co-location of PSD -95 was established at normal levels.

There is a correlation between the loss of synapses and the cognitive impairment generated in sepsis. Therefore, interventions such as the EE protocol can be beneficial in the synaptic plasticity of this pathology. It is important to emphasize that the limitation of this study is the choice of not using females.

## Conclusion

In conclusion, the physiological stress that is generated and transmitted to the immune system during severe sepsis, can have significant effects on gene expression in the brain, specifically in the hippocampus tissue, both in the short and long term. Because of this, EE may have the ability to delay and improve cognitive deficits induced by the disease and be a strategy with great potential for modulation of epigenetic enzymes in an animal model of sepsis.

## Data Availability

Material will available are request.

## Abbreviations

EE: Enriched environment
SE: Standard environmental condition
CLP: Cecal ligation and perforation
HAT: Histone acetylases
HDAC: Histone deacetylases
DNMT: DNA methyltransferases
PSD-95: Postsynaptic density protein 95
