# Author Correction: Enriched environment causes epigenetic changes in hippocampus and improves long-term cognitive function in sepsis

**DOI:** 10.1038/s41598-022-25622-3

**Published:** 2022-12-06

**Authors:** Emily Córneo, Monique Michels, Mariane Abatti, Andriele Vieira, Renata Casagrande Gonçalves, Filipe Fernandes Gabriel, Heloisa Borges, Amanda Goulart, Natan da Silva Matos, Diogo Dominguini, Roger Varela, Samira S. Valvassori, Felipe Dal‑Pizzol

**Affiliations:** 1 Laboratory of Experimental Pathophysiology, Graduate Program in Health Sciences, University of Southern Santa Catarina (UNESC), Av. Universitária, 1105, Criciúma, SC 88806-000 Brazil; 2 Translational Psychiatry Laboratory, Graduate Program in Health Sciences, University of Southern Santa Catarina (UNESC), Criciúma, SC Brazil

Correction to: *Scientific Reports* 10.1038/s41598-022-14660-6, published online 07 July 2022

The original version of this Article contained an error in one of the author names. Samira S. Valvassori was incorrectly given as Samira Valvassori.

The original Article has been corrected.

